# Production of α-1,3-L-arabinofuranosidase active on substituted xylan does not improve compost degradation by *Agaricus bisporus*

**DOI:** 10.1371/journal.pone.0201090

**Published:** 2018-07-24

**Authors:** Aurin M. Vos, Edita Jurak, Peter de Gijsel, Robin A. Ohm, Bernard Henrissat, Luis G. Lugones, Mirjam A. Kabel, Han A. B. Wösten

**Affiliations:** 1 Microbiology, Department of Biology, Utrecht University, Utrecht, The Netherlands; 2 Laboratory of Food Chemistry, Wageningen University and Research, Wageningen, The Netherlands; 3 Architecture et Fonction des Macromolécules Biologiques, Centre National de la Recherche Scientifique, Aix-Marseille Université, Marseille, France; Helsingin Yliopisto, FINLAND

## Abstract

*Agaricus bisporus* consumes carbohydrates contained in wheat straw based compost used for commercial mushroom production. Double substituted arabinoxylan is part of the ~40% of the compost polysaccharides that are not degraded by *A*. *bisporus* during its growth and development. Genes encoding α-1,3-l-arabinofuranosidase (AXHd3) enzymes that act on xylosyl residues doubly substituted with arabinosyl residues are absent in this mushroom forming fungus. Here, the AXHd3 encoding *hgh43* gene of *Humicola insolens* was expressed in *A*. *bisporus* with the aim to improve its substrate utilization and mushroom yield. Transformants secreted active AXHd3 in compost as shown by the degradation of double substituted arabinoxylan oligomers in an *in vitro* assay. However, carbohydrate composition and degree of arabinosyl substitution of arabinoxylans were not affected in compost possibly due to inaccessibility of the doubly substituted xylosyl residues.

## Introduction

*Agaricus bisporus* mushrooms are produced in the Netherlands using a wheat straw based compost. Wheat straw consists of 34–40% cellulose, 24–35% hemicellulose, and 14–24% lignin [1–4]. The hemicellulose fraction consists for a large part of glucuronoarabinoxylan. Its β-(1→4)-linked d-xylosyl backbone is decorated with l-arabinosyl, acetyl, and / or glucuronic acid residues that can be methylated and with d-galactosyl, rhamnose, and mannose as minor substituents [5,6]. Acetyl, glucuronic acid, and arabinosyl residues have a degree of substitution (based on xylosyl) of 0.1, 0.038, and 0.077, respectively. Some of the glucuronic acid is suggested to be linked to lignin via ester bonds [6], while the arabinosyl substituents can be covalently linked to lignin via ester or ether bonds with ferulic and coumaric acid [7–9].

Compost used by the Dutch *A*. *bisporus* industry is prepared in three phases using a mixture of wheat straw, horse manure, gypsum, and water, either or not supplemented with chicken manure as an additional nitrogen source [10,11]. During Phase I (PI), a mesophilic microbiota is replaced by a thermophilic microbiota with the substrate reaching a temperature of up to 80°C [10]. Lignin remains unaffected, while some xylan and cellulose is consumed [11–14]. In the conditioning process of phase II (PII) ammonia is sequestered by actinomycetes and thermophilic fungi like *Scytalidium thermophilum* (also named *Humicola insolens*; [15–17]). Lignin is still largely unaffected after PII, while 50% and 60% of cellulose and xylan have been removed, respectively [11,13]. Spawn of *A*. *bisporus* is introduced in PII-end compost initiating a 16–19 day colonization process at 25°C. Lignin is preferentially removed during this phase (PIII) with a loss of 50%, while only 15% and 10% of xylan and cellulose are degraded relative to PII, respectively [13]. Compost is transported to mushroom growers to start Phase IV (PIV) where it is topped with a casing layer. Mushrooms are produced in 2–3 flushes with a 7–8 day interval and a typical yield of 30 kg m^-2^ when using 85–95 kg compost m^-2^. In PIV an additional 44%, 29%, and 8% of cellulose, xylan, and lignin are degraded relative to PII, respectively [11,13]. It should be noted that the loss of cellulose in PI–PIV is underestimated since glucose originating from cellulose cannot be distinguished from fungal glucan. Still, this leaves a significant proportion of the carbohydrates unutilized for mushroom production.

Recalcitrant hemicellulose accumulates as xylan substituted with (4-*O*-methyl) glucuronic acid and single and double substitutions of arabinosyl residues [18,19]. The arabinosyl and glucuronoyl substitution ratio of xylose increases during PIV but not during PIII of mushroom cultivation [12,18]. The genome of *A*. *bisporus* encodes GH115 α-glucuronidase genes (geneID 121649 and 121650; [20]). Based on characterization of other GH115 proteins it is expected that those of *A*. *bisporus* act on (4-*O*-methyl) glucuronic acid substituted xylan [21]. The encoded proteins are actively produced in beech wood xylan [22], but their activity could not be found in compost [18,23]. The genome of *A*. *bisporus* contains one GH51 α-l-arabinofuranosidase that may be active on single and, possibly, doubly substituted arabinosyl residues [24]. However, it is not clear if this enzyme is produced since no α-l-arabinofuranosidase activity was found in compost extract [25]. Furthermore, the GH51 would only be active on the external double substitutions present on the non-reducing end of arabinoxylan. GH43 α-l-arabinofuranosidases predicted to act on the *O*3 position of internal and terminal doubly substituted xylosyl residues (AXHd3) are absent in the genome of *A*. *bisporus* [19], which would explain why arabinose substituents accumulate in compost. The fact that AXHd3 of *Bifidobacterium adolescentis* could cleave doubly substituted xylosyl residues in KOH–xylan-extracts from compost collected after the 2^nd^ flush supports the absence of α-1,3-l-arabinofuranosidase activity from *A*. *bisporus* [25]. Here, the AXHd3 encoding *hgh43* gene from *Humicola insolens* was introduced in *A*. *bisporus* to assess whether this activity increases substrate utilization by this mushroom forming fungus. Although transformants produced active enzyme, no effect on carbohydrate composition or xylan substitution was found during PIII and PIV. This indicates that AXHd3 activity is not the bottleneck to improve compost degradation.

## Material and methods

### Strains and substrate

*Agaricus bisporus* strain A15 (Sylvan, Netherlands) was routinely grown at 25 ˚C on malt extract agar medium (MEA; 20 gr l^-1^ malt extract [BD biosciences, Franklin Lakes, USA], 1.5% agar, 2.1 gr l^-1^ MOPS, pH 7.0). Mycelium for RNA isolation was grown for 16 days from 4 mycelial plugs (5 x 5 mm) on a polycarbonate (PC) membrane (diameter, 76 mm; pore size, 0.1 μm) overlaying MEA.

Spawn was made by inoculating Erlenmeyers containing a mixture of 50 g rye, 1.4 g CaCO_3_, 1 g CaSO_4_, and 50 ml demi water with 0.5 by 0.5 cm pieces of colonized MEA. After 3 weeks of growth, spawn was stored at 4°C. For small scale cultivation of *A*. *bisporus* 20 g spawn was mixed with 2.5 kg PII compost (CNC Grondstoffen, Milsbeek, the Netherlands) in a box (30 x 20 x 22 cm) overlaid with plastic foil containing 20 evenly distributed holes of 2–3 mm. Three boxes were used per strain. After 16 days of growth at 24°C, the compost was topped with 1 kg casing layer (CNC Grondstoffen, Milsbeek, the Netherlands) and growth was prolonged for 7 days. Plastic foil containing 20 evenly distributed holes of 2–3 mm prevented water evaporation. Mushroom formation was induced by removing the foil and lowering the air temperature to 20°C. Mushrooms were harvested in two flushes during a 2 week period.

### Plasmid construction

The coding sequence of *H*. *insolens hgh43* (GenBank: CAL81199.1) was codon optimized using the OptimumGeneTM algorithm (Genscript USA Inc). To this end, a codon usage table of coding sequences of *A*. *bisporus* (strain H97, genome version 2.0) was used combined with that of the 1000 most highly expressed genes on compost [20]. The codon tables were produced using a custom Python script that discarded coding sequences that did not start with ATG or did not end with an in frame stop codon. The codon optimized α-1,3-l-arabinofuranosidase gene was ordered at Genscript (New Jersey, United States). The gene contained intron 4 of *gpdII* (gene ID 138631) including 6 upstream and 8 downstream nucleotides after its stop codon. To create the expression vector, the actin promoter and terminator of *A*. *bisporus* were amplified with primers 1 & 2 and 3 & 4 (S1 Table). Fragments were cloned in pGEMt and reamplified with primers 5 & 6 and 7 & 8 (S1 Table). They were cloned in PacI / AscI digested pBHg-PA [26] using InFusion cloning, resulting in pBHg-ActPT. Primers 9 & 10 were used to amplify codon optimized *hgh43* with the intron following the stop codon using Phusion polymerase (S1 Table). InFusion cloning (Takara Bio USA, Inc) was used to introduce the amplified fragment in between the 5’ and 3‘ actin regulatory elements in PacI / AscI digested pBHg-ActPT resulting in plasmid pBHg-HGH43.

### Transformation of *Agaricus bisporus*

Plasmid pBHg-HGH43 was transformed to *A*. *bisporus* A15 gills using *Agrobacterium tumefaciens* mediated transformation [27]. For selection, gills were placed on MEA containing 25 μg ml^-1^ hygromycin, 200 μM cefotaxime, and 100 μg ml^-1^ chloramphenicol. Resistant mycelium originating from gills was transferred to a second selection plate containing 40 μg ml^-1^ hygromycin.

### RNA isolation and qPCR

Mycelium was homogenized for 1 min with 2 metal balls at 25 Hz in a 2 ml tube that was placed in a holder cooled to -80°C. RNA was extracted with 500 μl Trizol reagent [28]. After a 5 min incubation at room temperature, 200 μl chloroform was added and phases were separated through centrifugation at 15000 g for 15 min. RNA in the aqueous phase was precipitated by addition of 0.5 volume isopropanol and centrifugation at 15000 g for 15 min. RNA was washed with 70% ethanol and dissolved in water. cDNA was prepared from 1 μg of RNA using the Quantitect® reverse transcription kit (QIAGEN).

An optical 96 wells plate (Applied Biosystems) and a ViiA™ 7 Real-Time PCR System (Thermo Fisher Scientific) were used for qPCR with SYBR® Green to monitor DNA synthesis. Primers 11 & 12, 13 & 14, and 15 & 16 (S1 Table) were used to detect *gpdII*, *18S*, and *hgh43*, respectively. Reactions were performed using 40 cycles of 15 s at 95°C and 1 min at 60°C preceded with an incubation for 2 min at 50°C and 10 min at 95°C. RNA levels of *hgh43* were calculated relative to *gpdII* and *18S* using the 2^−ΔΔCt^ method [29,30].

### α-l-arabinofuranosidase assay

An aliquot of 10 g compost harvested after the 2^nd^ flush was mixed with 100 ml water and shaken for 1 h at 250 rpm. Compost particles were removed by centrifugation for 15 min at 4500 *g*, after which the extract was filter sterilized (Minisart® syringe filter, 0.45 μm), concentrated, and washed with 4 ml ultra-pure water using Amicon Ultra-4 centrifugal filters (Merck Millipore, Billerica, USA) with a pore size of 10 kDa. Protein concentrations were measured using a Pierce™ BCA Protein Assay Kit (Thermoscientific) according to the manufacturers instruction (S2 Table). Concentrated extract (150 μl) was supplemented with wheat arabinoxylan (WAX) oligomers (45 μl, 2.5 mg ml^-1^ in 20 mM NaOAc, pH 4.5), 0.1% azide (5 μl, 8 mg ml^-1^), and incubated overnight at 37°C. The WAX oligomers were prepared by treating WAX (medium viscosity wheat arabinoxylan, Megazyme, Ireland) with a pure and well characterized endo-xylanase [31]. High performance anion exchange chromatography (HPAEC) was performed to analyze reaction mixtures. To this end, a Dionex ICS-5000 unit (Dionex, Sunnyvale, USA) equipped with a CarboPac PA-1 column (2 mm x 250 mm ID) was used in combination with a CarboPac guard column (2mm x 50 mm ID) and pulsed amperometric detection (PAD). Chromelion software (Thermo scientific, Sunnyvale, USA) was used to control the system. Flow rate during the 35-min elution was 0.3 mL min^-1^ using a linear gradient from 0–38% 1 M NaOAc in 0.1 M NaOH. A 3 min cleaning step with 100% 1 M NaOAc in 0.1 M NaOH and a 12 min equilibration step with 0.1 M NaOH were used in between runs. Identification and quantification of mono- and oligosaccharides was not affected by compounds present in the compost extracts.

### Analysis of neutral sugars and uronic acids

Lyophilized compost samples were incubated for 1 h at 30°C in 72% (w / w) H_2_SO_4_, after which samples were hydrolyzed for 3 h in 1 M H_2_SO_4_ at 100°C. Alditol acetate derivatives of the sugars were produced and analyzed using gas chromatography (FocusGC, Thermo Scientific, Waltham, USA) using inositol as internal standard [32].

Uronic acid content was measured as anhydro-uronic acid using an automated m-hydroxydiphenyl assay [33] with addition of sodium tetraborate using an autoanalyzer (Skalar Analytical, Breda, The Netherlands). Glucuronic acid (12.4 to 200 μg mL^-1^; Fluka AG, Busch, Switzerland) was used as a reference. The sum of neutral sugars and uronic acids was defined as the total carbohydrate content.

### Identification of the GH43_36 subfamily

The assignment of sequences to GH43_36 was done by HMMer3 [34] search against the collection of HMMs developed for each GH43 subfamily defined in Mewis et al. (2016) [35], and verified by BLAST analysis [36] against the sequences of the CAZy database (www.cazy.org; [37]) already classified in GH43_36.

## Results

### Phylogeny of GH43_36

**Fig 1:**
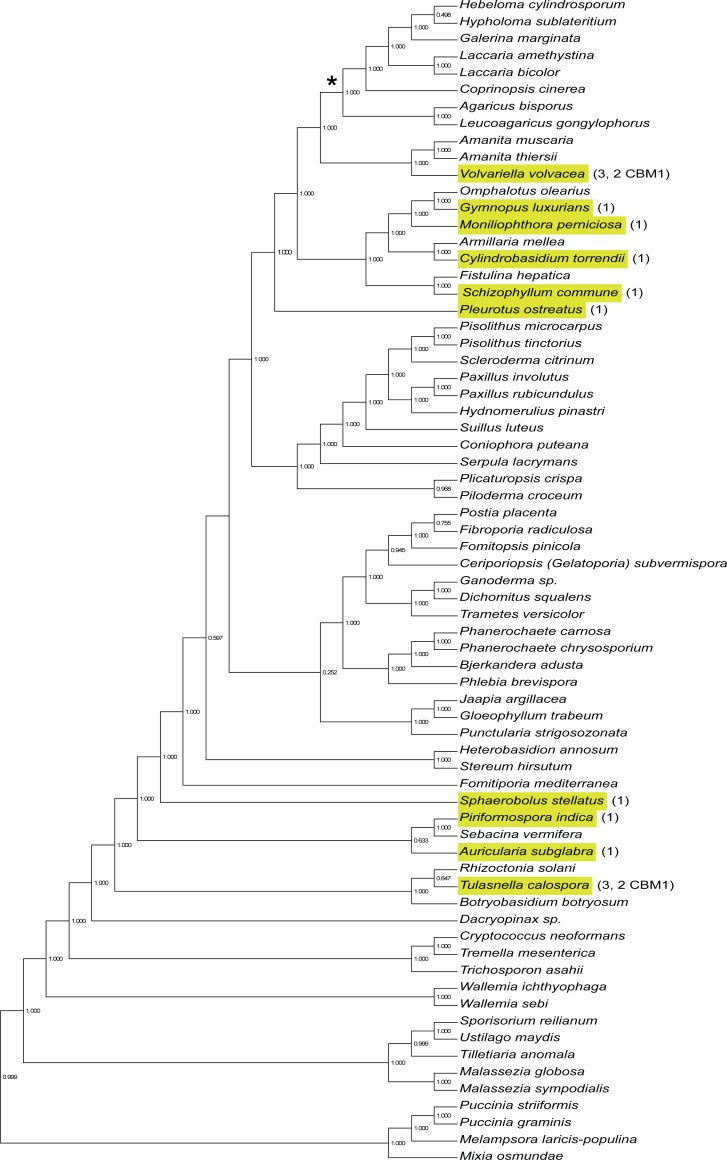
GH43_36 distribution in Basidiomycota. Phylogenetic tree of 69 basidiomycetes based on 71 highly conserved fungal genes. Presence of genes of the GH43_36 subfamily are indicated in yellow. The number of genes and the presence of CBM1 domains within the proteins are indicated with numbers. Inferred loss of GH43_36 in the ancestor of *A*. *bisporus* is indicated with an asterisk. Adapted and modified from [35].

To introduce AXHd3 activity in commercial *A*. *bisporus* strains via conventional breeding it is necessary for this trait to be present in the wild type population. To predict its presence, the distribution of genes of this subfamily was assessed in 145 fungal genomes [35] (S3 Table), of which the clade with 69 basidiomycetes is presented in Fig 1. The GH43_36 subfamily appears to be present in two clusters of basidiomycetes and is absent in the order *Agaricales* that includes *A*. *bisporus*. The GH43 proteins of *A*. *bisporus* were previously identified as GH43_5 arabinases (protein ID 224152 and 119499), GH43_13 bifunctional xylosidase / α-l-arabinofuranosidase (protein ID 211524), and a protein that is part of the uncharacterized GH43_23 subfamily (protein ID 208425) [19,34].

### Introduction of *hgh43* in *A*. *bisporus*

**Fig 2:**
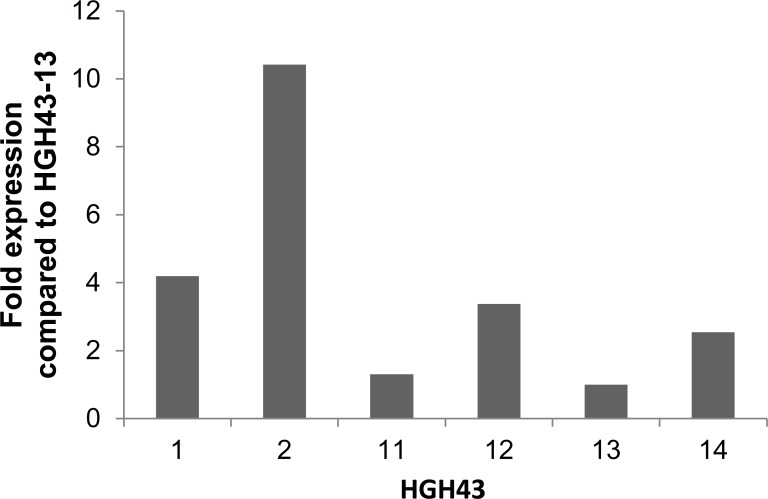
Expression of *hgh43* in *A*. *bisporus* transformants relative to transformant HGH43-13.

The coding sequence of the AXHd3 gene *hgh43* of *H*. *insolens* was codon optimized for expression in *A*. *bisporus* and introduced in this basidiomycete under control of *A*. *bisporus* actin regulatory sequences. Expression of this gene was assessed by qPCR in MEA grown cultures of 6 transformants. As expected no *hgh43* expression was found in wild-type A15. Transformants HGH43-1 and HGH43-2 expressed *hgh43* most highly with a 4- and 10- fold higher expression as compared to HGH43-13 when normalized to *gpdII* and *18S* (Fig 2).

### Small scale cultivation of *A*. *bisporus* expressing *hgh43*

Strains HGH43-1 and HGH43-2 were selected for a small scale cultivation to assess carbohydrate degradation and GH43 activity in compost. To this end, PII compost was mixed with spawn of A15 wild type or strains HGH43-1 and HGH43-2. After 16 days of compost colonization, PIV was initiated by topping the compost with casing layer. Mushrooms were harvested in two flushes. Total fresh weight of mushrooms of strain A15 was 293 g kg^-1^ compost. The mushroom yield of the transformants was 80–100% of that of A15.

**Fig 3:**
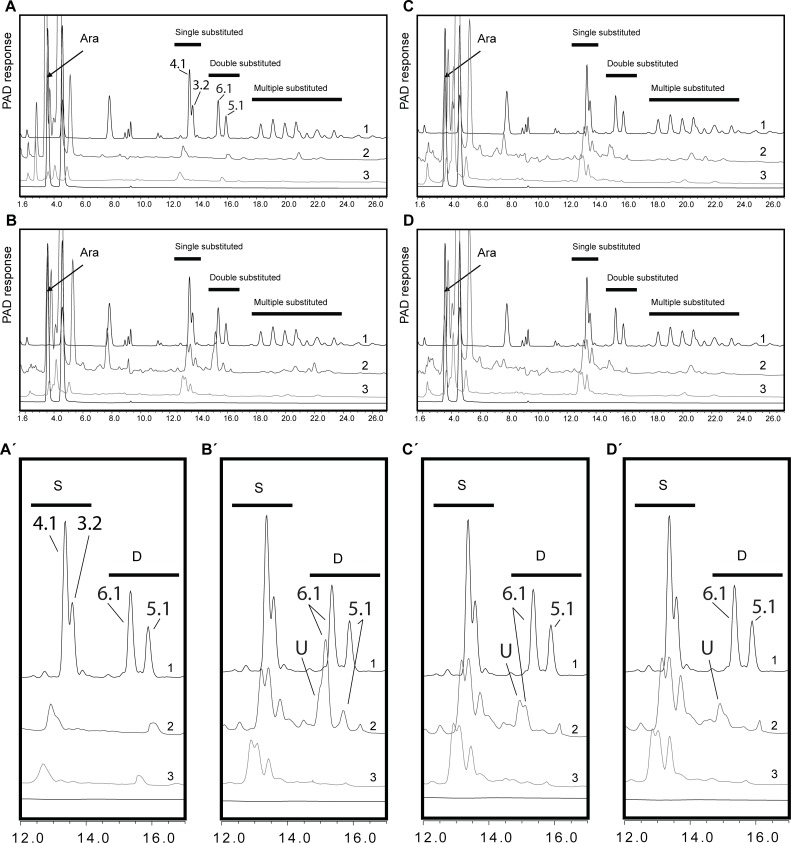
HPAEC chromatogram of endoxylanase pre-digested WAX incubated with concentrated compost extract. PII extract (A) and extract of compost colonized by A15 (B), HGH43-1 (C), and HGH43-2 (D). Endoxylanase pre-digested WAX without compost extracts (1), with concentrated compost extract (2), and compost extract without WAX-oligomers added (3) are shown in each panel. The line closest to the x-axis shows the retention time of arabinose and xylose at 3.5 and 4.5 min, respectively. Each sample is representative for a biological triplicate. The chromatogram is magnified from retention time 12–16.5 min (A’, B’, C’, and D’) for each sample where single and double substituted arabinoxylan oligomers are indicated with S and D, respectively. Specific xylosyl oligomers substituted with a single arabinosyl are indicated with 4.1, 3.2 in A and A’. Xylosyl oligomers with a double arabinosyl substitution are indicated with 6.1 and 5.1 in A, A’, B’, C’, and D’. An unknown oligomer is indicated with U in B’, C’, and D’. Chromatograms of compost extract or compost extract incubated with WAX were shifted 10–30 sec to the left as compared to endoxylanase hydrolyzed WAX.

Oligomers of wheat arabinoxylan (WAX) substituted with arabinosyl residues were incubated with compost extracts and analyzed by HPAEC (Fig 3; S1 Fig; S2 Fig; S2 Table). Compost extract from PII-end compost (i.e. before inoculation with *A*. *bisporus*) completely degraded both single and double arabinosyl substituted WAX oligomers (Fig 3A and 3A’). Single arabinosyl substituted xylan oligomers (Fig 3, S1 Fig; structure 3.2 and 4.1) were also degraded when WAX-oligomeric substrate was incubated with PIV compost extracts of A15 and its transformants (Fig 3B’–3D’, 13.15 and 13.30 min). In addition, these extracts degraded the multiple substituted oligomers (S1 Fig; structures 6.2, 5.2, 8.1, 7.1, 10.1, 6.3, and 9.1) almost completely (Fig 3B–3D, 18–24 min). The doubly substituted oligomer 5.1 decreased in abundance in WAX-oligomeric substrate treated with A15 compost extract (Fig 3B’–3D’, 15.45 min). Conversely, the double substituted oligomer 6.1 increased in abundance (Fig 3B’, 15.15 min). In addition, an unknown oligomer with a slightly lower retention than 6.1 had accumulated (Fig 3B’, shoulder of the 6.1 peak indicated with U). Compost extract from the *hgh43* transformants was more active on the 5.1 oligomer as compared to compost extract from A15 (Fig 3C’–3D’, 15.45 min). Furthermore, the abundance of the 6.1 oligomer and the unknown oligomer that eluted between 14.45 and 15.30 min was up to 73% lower in the transformants as compared to A15 (Fig 3C’ and 3D’; T-test, p < 0.05). The average area of A15, GH43-1, and HGH43-2 was 11.2, 4.5, and 3.0 nC min, respectively, with standard deviations of 1.6, 1.7, and 0.4, respectively. Together, this shows that α-l-arabinofuranosidase active on doubly substituted xylosyl residues was produced by *hgh43* transformants and that this activity is significantly lower in the extracts of A15 colonized compost.

Carbohydrate compositions of PII-end compost and PIV compost were analyzed (Table 1) to assess the impact of *hgh43* expression during cultivation of *A*. *bisporus*. The relative abundance of glucosyl, xylosyl, and arabinosyl residues in PII-end compost was 56.1, 30.3, and 4.3% (mol / mol), respectively. Relative abundance of glucosyl and xylosyl (Xyl) residues had decreased to 51.3 and 26.6% (mol / mol), respectively, while the abundance of arabinosyl (Ara) residues had increased to 4.8% (mol / mol) after 2 flushes of A15 mushroom production. As a consequence, the Ara / Xyl ratio had increased from 14 to 18 (mol / 100 mol) in PII-end compost and after the 2^nd^ flush, respectively. No differences in carbohydrate composition (mol / mol %) or Ara / Xyl ratio were observed between A15 and the strains producing AXHd3.

**Table 1:** Carbohydrate composition (mol / mol %) of compost.

Compost	Rha	Man	Gal	Glc	Uronic acid	Ara	Xyl	Ara/Xyl (mol / 100 mol)
**PII**	0.7 (0.07)	1.6 (0.22)	1.5 (0.14)	56.1 (0.14)	5.3 (0.28)	4.3 (0.19)	30.3 (0.77)	14.1 (1.01)
**PIV A15**	1.1 (0.13)	6 (0.66)	2.2 (0.09)	51.3 (1.51)	7.7 (0.65)	4.8 (0.39)	26.6 (0.98)	18.1 (1.19)
**PIV HGH43-1**	1.2 (0.1)	6.3 (0.81)	2.5 (0.27)	50.2 (0.37)	8 (0.41)	4.9 (0.29)	26.5 (1.45)	18.8 (2.02)
**PIV HGH43-2**	1.2 (0.16)	6.3 (0.48)	2.3 (0.08)	51.6 (1.15)	8 (0.54)	4.9 (0.21)	25.5 (0.73)	19.2 (0.35)

PII compost and PIV compost colonized by strains A15, HGH43-1, and HGH43-2. Averages and standard deviation (in parentheses) of biological triplicates are shown.

## Discussion

Compost colonization and subsequent mushroom production by *A*. *bisporus* has been proposed to be limited by the inability of this fungus to degrade recalcitrant polysaccharides such as doubly substituted arabinoxylan [18,19]. AXHd3 removes the *O*3 linked arabinosyl decorations from the xylan backbone. Phylogenetic analysis revealed that 59 out of 69 basidiomycetes did not have a predicted AXHd3 gene, among which mushroom forming basidiomycetes such as *A*. *bisporus*, *Coprinopsis cinerea*, and *Laccaria bicolor*. Therefore, it is unlikely that natural strains of *A*. *bisporus* will contain this gene. The absence of AXHd3 genes in *A*. *bisporus* could explain why arabinoxylan accumulates during cultivation of this mushroom forming fungus.

*H*. *insolens* colonizes compost during PII before the introduction of *A*. *bisporus* [38]. The GH43 AXHd3 of *H*. *insolens* acts specifically on the *O*3 position of doubly substituted xylosyl residues [39] and would thereby be involved in removing doubly substituted arabinoxylan during this stage of composting. Indeed, α-l-arabinofuranosidase contained in PII compost extract degraded both single and double substituted arabinoxylo-oligosaccharides (Fig 3A). Previously it was shown that double substitutions were not removed by compost extract of PIII and PIV compost [20]. As a solution, the AXHd3 gene *hgh43* of *H*. *insolens* was introduced in *A*. *bisporus* controlled by actin regulatory elements. The pH optimum of the encoding enzyme, pH 6.7 [39], is close to that found in PIII and PIV compost (being 7 and 6.5, respectively). AXHd3 activity was found in PIV compost colonized by *hgh43* transformants and significantly lower in extract of compost colonized by A15. This low activity may be derived from A15 or from other microbes in the compost such as from *H*. *insolens*.

Enzyme activities in compost extract from A15 were able to degrade multiple substituted arabinoxylo-oligomers. This is explained by xylanases that cleave the large oligomers into smaller single and double substituted arabinoxylo-oligomers. The small single substituted arabinoxylan oligomers were also completely degraded but not the doubly substituted oligomers. Part of the double substituted 5.1 structure disappeared by the action of A15 compost extract. The latter may be explained by yet unknown enzymes produced by *A*. *bisporus* active on these double substituted residues. Alternatively, it may be caused by GH51 α-l-arabinofuranosidase activity that acts on xylan with double substituted arabinose residues at the non-reducing terminal xylose [24,40–42]. *A*. *bisporus* contains one GH51 that is highly expressed during its vegetative growth but less active during mushroom formation [23]. This would agree with the lack of arabinosyl accumulation during PIII [18]. Structure 5.1 may also (partly) disappear due to the action of a xylanase that removes the terminal non-substituted xylose from this oligomer that consists of a backbone of 3 xylose residues. This may explain the appearance of an unknown oligomer eluting slightly faster than structure 6.1 that was formed when arabinoxylo-oligomers were incubated with compost extract from A15. Together, it is clear that compost extracts from the *hgh43* transformants more actively remove doubly substituted arabinoxylo-oligomers than A15.

The production of AXHd3 by *A*. *bisporus* was expected to result in a reduction of the degree of substitution (DS) of xylan in colonized compost by removal of arabinose from double substituted arabinoxylan. Consequently, a reduction in the Ara / Xyl ratio was expected. However, no difference in carbohydrate composition or Ara DS was found in compost colonized by the AXHd3 producing transformants as compared to compost colonized by A15. This may be explained by inaccessibility of the double substituted arabinoxylan by hemicellulose-lignin crosslinks [43,44]. Improved ligninolysis may therefore be required to benefit from AXHd3 production. Overexpression of manganese peroxidase in *A*. *bisporus* however did not result in improved lignin degradation possibly due to limited extracellular generation of the cofactor H_2_O_2_ [45].

Improved degradation of compost during mushroom cultivation will reduce the amounts of spent compost. Yet, it may not result in increased mushroom production. Cellulose and hemicellulose supplemented to PIII compost was degraded but did not increase mushroom yield [46]. This suggests that mushroom formation by this basidiomycete is not limited by extracellular enzymatic activity, and thereby sugar acquisition, but by other factors such as the differentiation state of the vegetative mycelium.

## Supporting information

S1 Table Primers used in this study. (DOCX)

S2 Table Protein concentrations in compost extracts. (XLSX)

S3 Table Overview of GH43_36 in 145 fungal species. (XLSX)

S1 Fig HPAEC elution profiles of WAX digested with endoxylanase (EX1; based on [31,47]). Single and double arabinoxylan oligomers and oligomers with both single and double substitutions are schematically represented. Ovals represent xylosyl residues and diamonds represent arabinosyl residues.
(EPS)

S2 Fig All replicates of HPAEC chromatograms of endoxylanase predigested WAX incubated with concentrated compost extract. (PPTX)
