# Supplementary material for: Production of α-1,3-L-arabinofuranosidase active on substituted xylan does not improve compost degradation by Agaricus bisporus
Source: PLoS One. 2018 Jul 24;13(7):e0201090. doi: 10.1371/journal.pone.0201090 (PMC6057652; doi:10.1371/journal.pone.0201090)
Supplement: S1 Table — (DOCX) [file pone.0201090.s001.docx]

**Table S1. Primers used in this study.**

| **Number** | **Name** | **Sequence (5' - 3')** |
| --- | --- | --- |
| 1 | Actin prmtr F | AAGCTTAGCCGAGAGAAGATGCCCC |
| 2 | Actin prmtr R | CCATGGTTTGTTATTCGTGTGTTCG |
| 3 | Actin trmntr F | GGATCCGCTGATGGTGCTTTATGATAAATAAAGTCCTTGGG |
| 4 | Actin trmntr R | GAATTCTACTACTACCCCCAAAACCGACATCATCC |
| 5 | Act-Pr_F | CCAGGGGGATCGTTAAAGCTTAGCCGAGAGAAG |
| 6 | pBHg_ActP_R | AATTAAGAATTCAGATCTCAATTGGGCGCGCCTTTGTTATTCGTGTGTTCG |
| 7 | pBHg_ActT_F | TCTGAATTCTTAATTAAGGATCCGCTGATGGTGCTTTATG |
| 8 | Act-Ter_R | CGCCGAATTGGCGCGGAATTCTACTACTACCCCC |
| 9 | HiGH43_F | AATAACAAAGGCGCGATGCTCGGACTTAAAGTTTTG |
| 10 | HiGH43_R | ATCAGCGGATCCTTAGATTGAACGGGCTGCCATG |
| 11 | APqGPD_F | TCGATCTTGTTGTTCGTCTTGAG |
| 12 | APqGPD_R | GCGCATGACCTCCTTGATTT |
| 13 | q18S_F | TCGCCGCTCCCTTGGT |
| 14 | q18S_R | GCATCGCCGGCACAA |
| 15 | qPCR_HiGH43_F | ATCGGCATGGAATTGGCAAC |
| 16 | qPCR_HiGH43_R | AGAGTTGCGCAACGTTGATG |
