# Supplementary material for: Production of α-1,3-L-arabinofuranosidase active on substituted xylan does not improve compost degradation by Agaricus bisporus
Source: PLoS One. 2018 Jul 24;13(7):e0201090. doi: 10.1371/journal.pone.0201090 (PMC6057652; doi:10.1371/journal.pone.0201090)
Supplement: S2 Fig — (PPTX) [file pone.0201090.s005.pptx]

## Slide 1
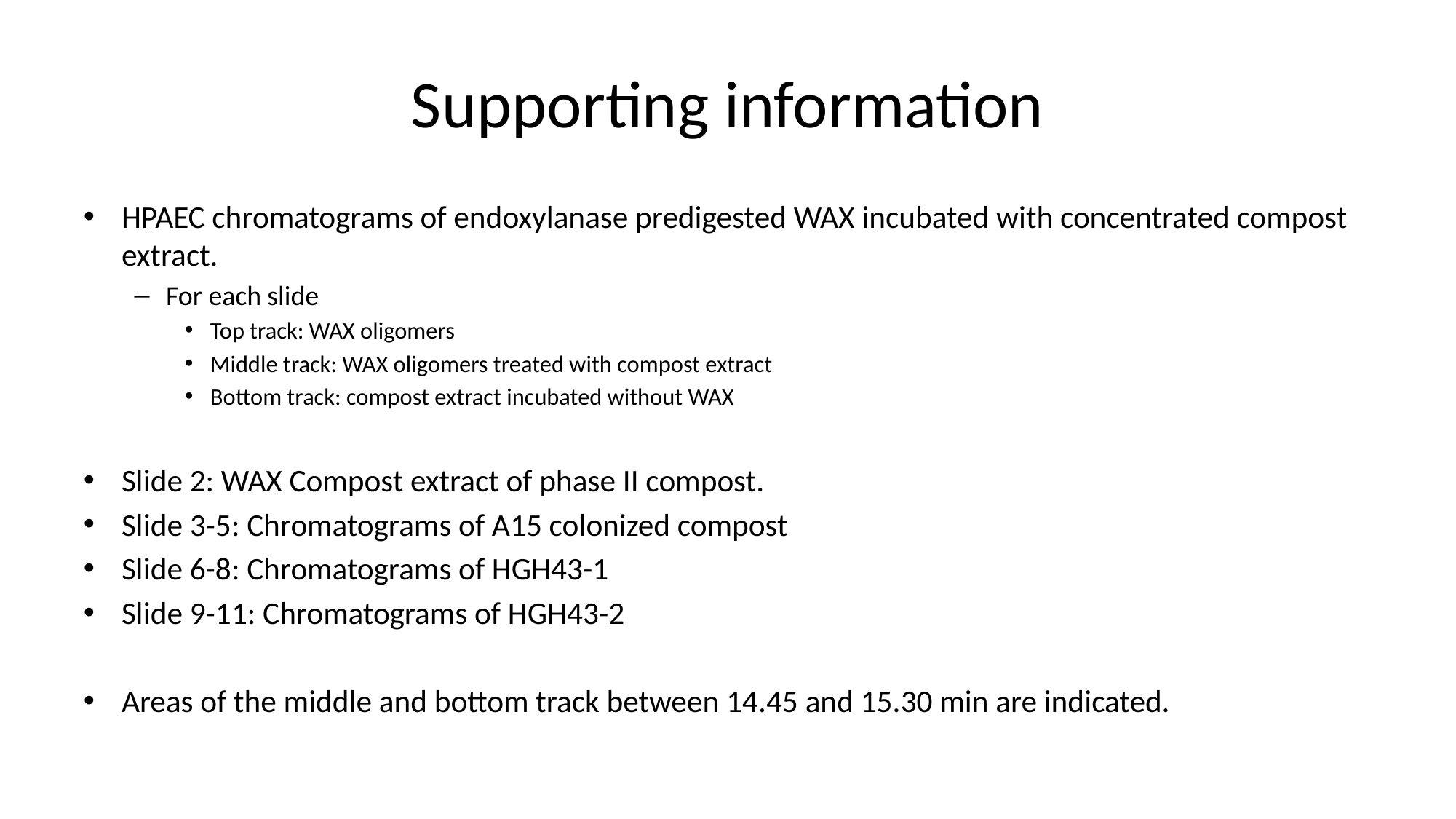

# Supporting information
HPAEC chromatograms of endoxylanase predigested WAX incubated with concentrated compost extract.
For each slide
Top track: WAX oligomers
Middle track: WAX oligomers treated with compost extract
Bottom track: compost extract incubated without WAX
Slide 2: WAX Compost extract of phase II compost.
Slide 3-5: Chromatograms of A15 colonized compost
Slide 6-8: Chromatograms of HGH43-1
Slide 9-11: Chromatograms of HGH43-2
Areas of the middle and bottom track between 14.45 and 15.30 min are indicated.

## Slide 2
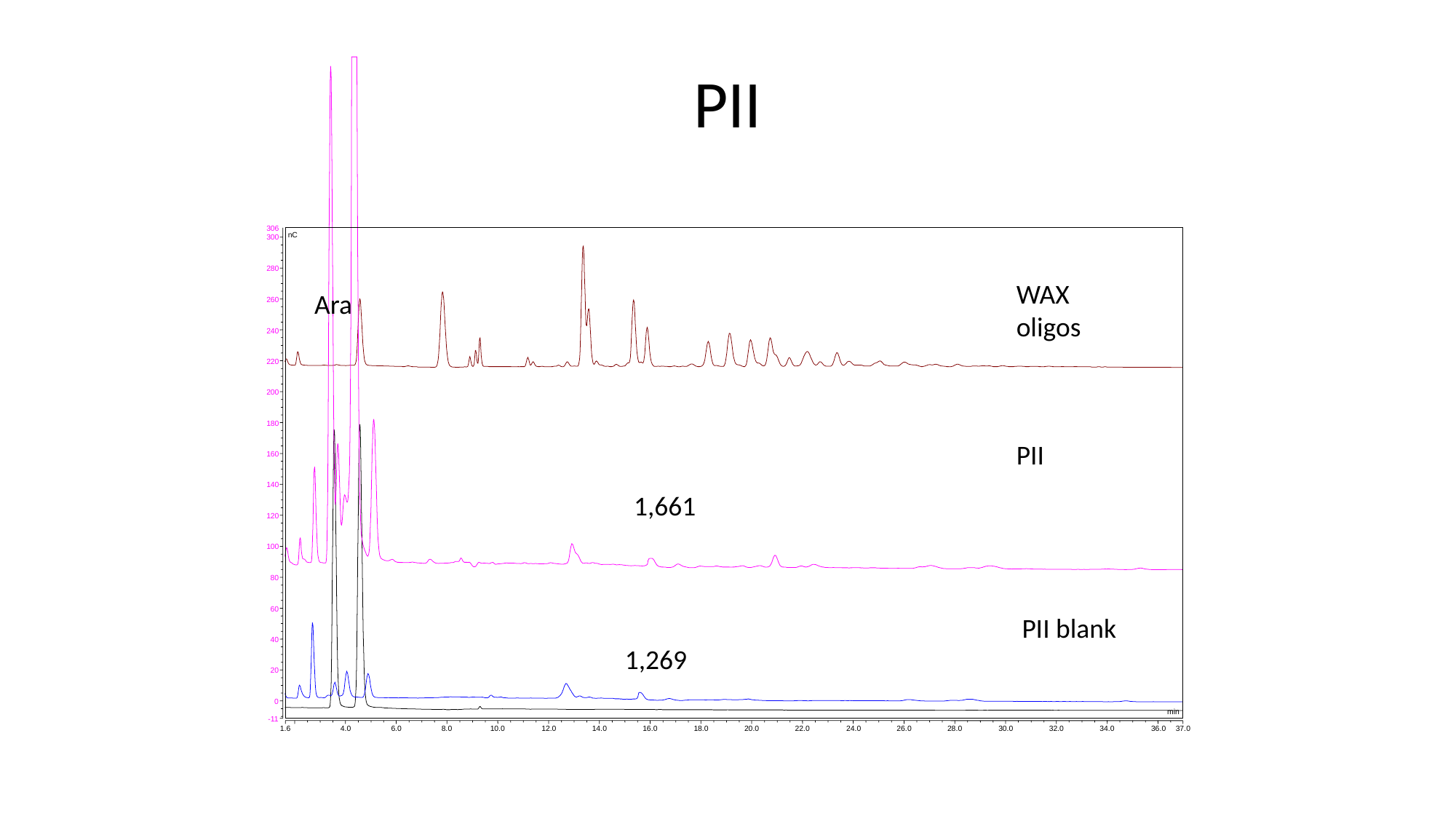

# PII
306
nC
300
280
WAX oligos
Ara
260
240
220
200
180
PII
160
140
1,661
120
100
80
60
PII blank
40
1,269
20
0
min
-11
1.6
4.0
6.0
8.0
10.0
12.0
14.0
16.0
18.0
20.0
22.0
24.0
26.0
28.0
30.0
32.0
34.0
36.0
37.0

## Slide 3
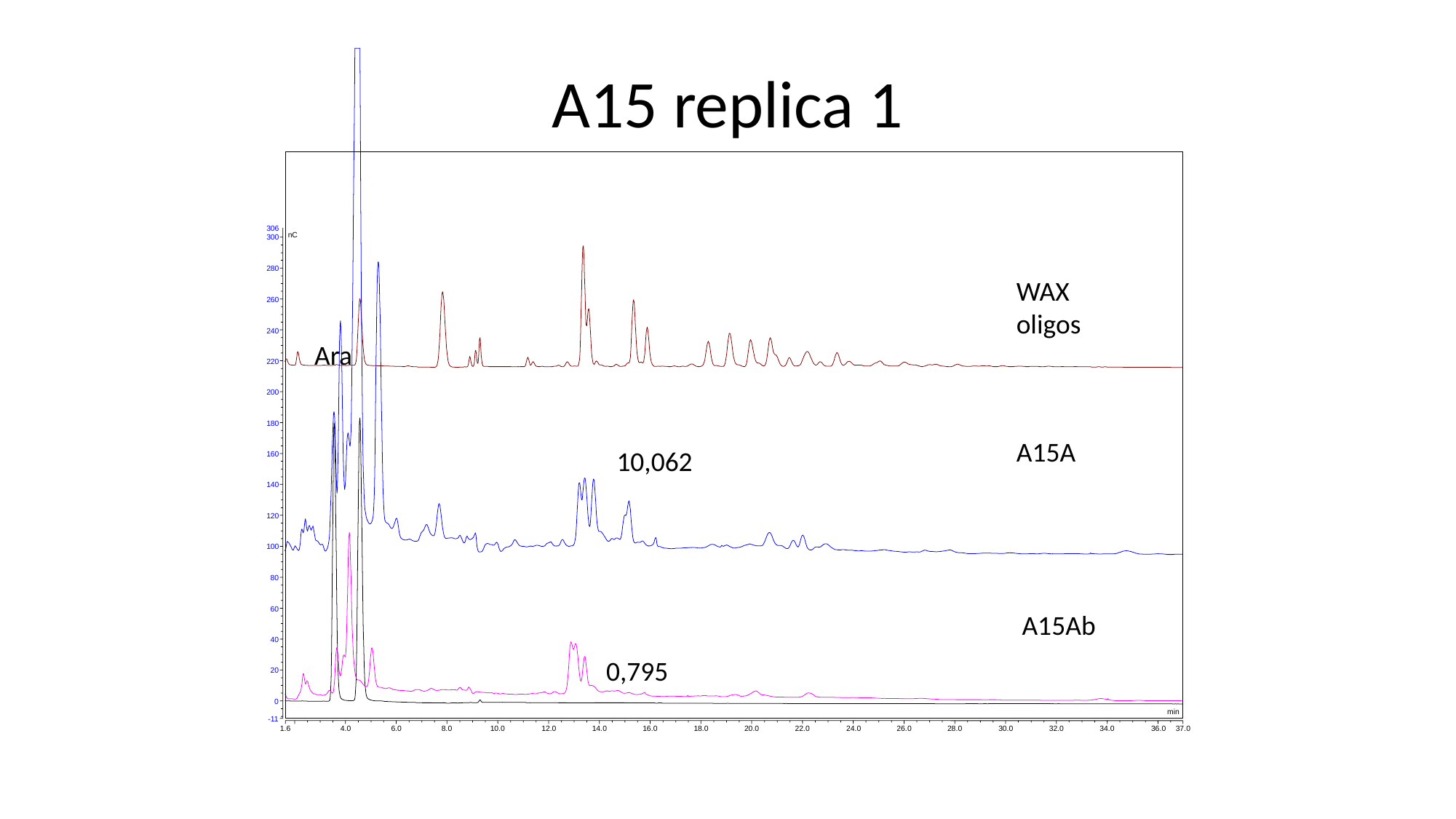

# A15 replica 1
306
nC
300
280
WAX oligos
260
240
Ara
220
200
180
A15A
10,062
160
140
120
100
80
A15Ab
60
40
0,795
20
0
min
-11
1.6
4.0
6.0
8.0
10.0
12.0
14.0
16.0
18.0
20.0
22.0
24.0
26.0
28.0
30.0
32.0
34.0
36.0
37.0

## Slide 4
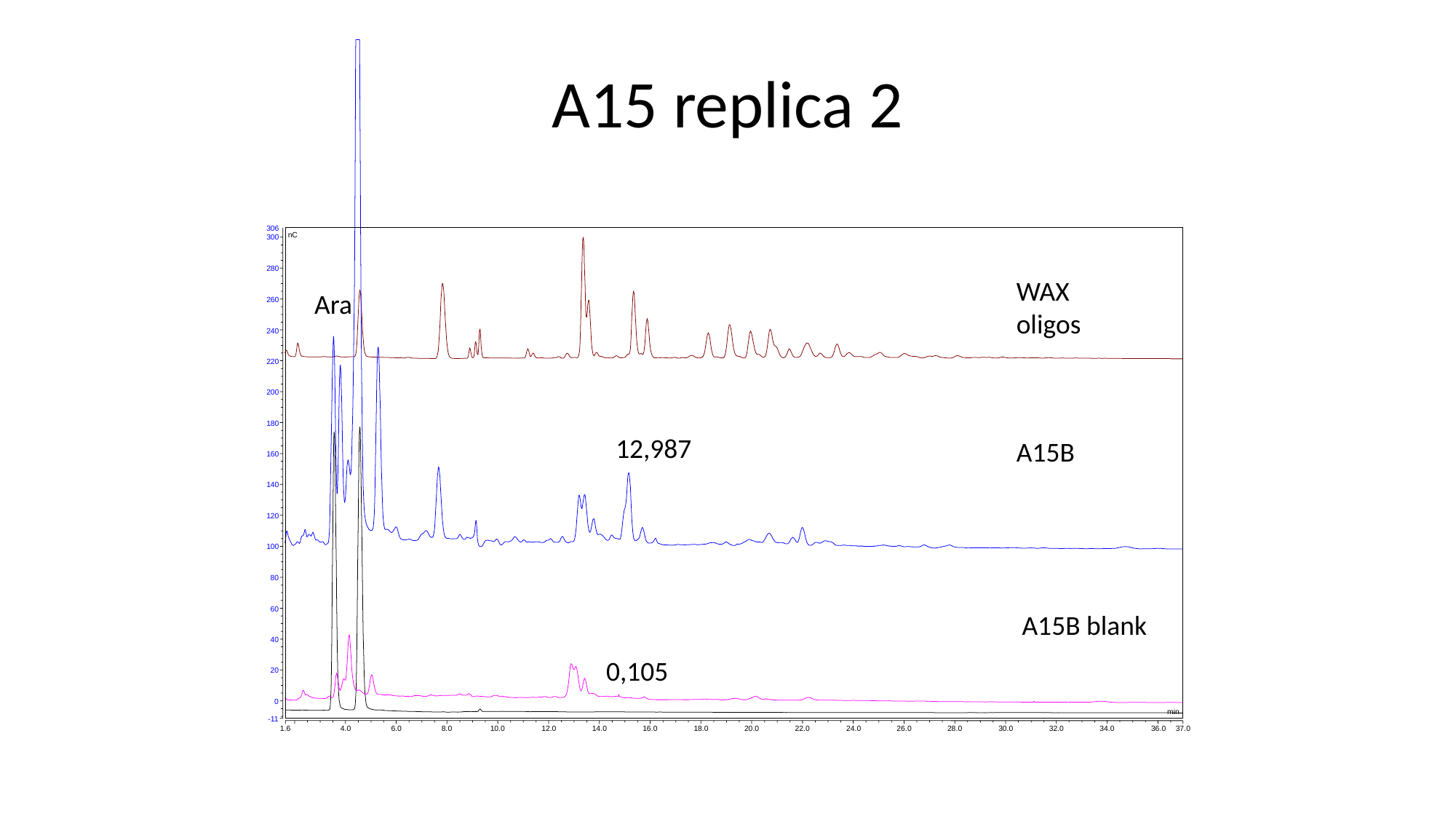

# A15 replica 2
306
nC
300
280
WAX oligos
Ara
260
240
220
200
180
12,987
A15B
160
140
120
100
80
A15B blank
60
40
0,105
20
0
min
-11
1.6
4.0
6.0
8.0
10.0
12.0
14.0
16.0
18.0
20.0
22.0
24.0
26.0
28.0
30.0
32.0
34.0
36.0
37.0

## Slide 5
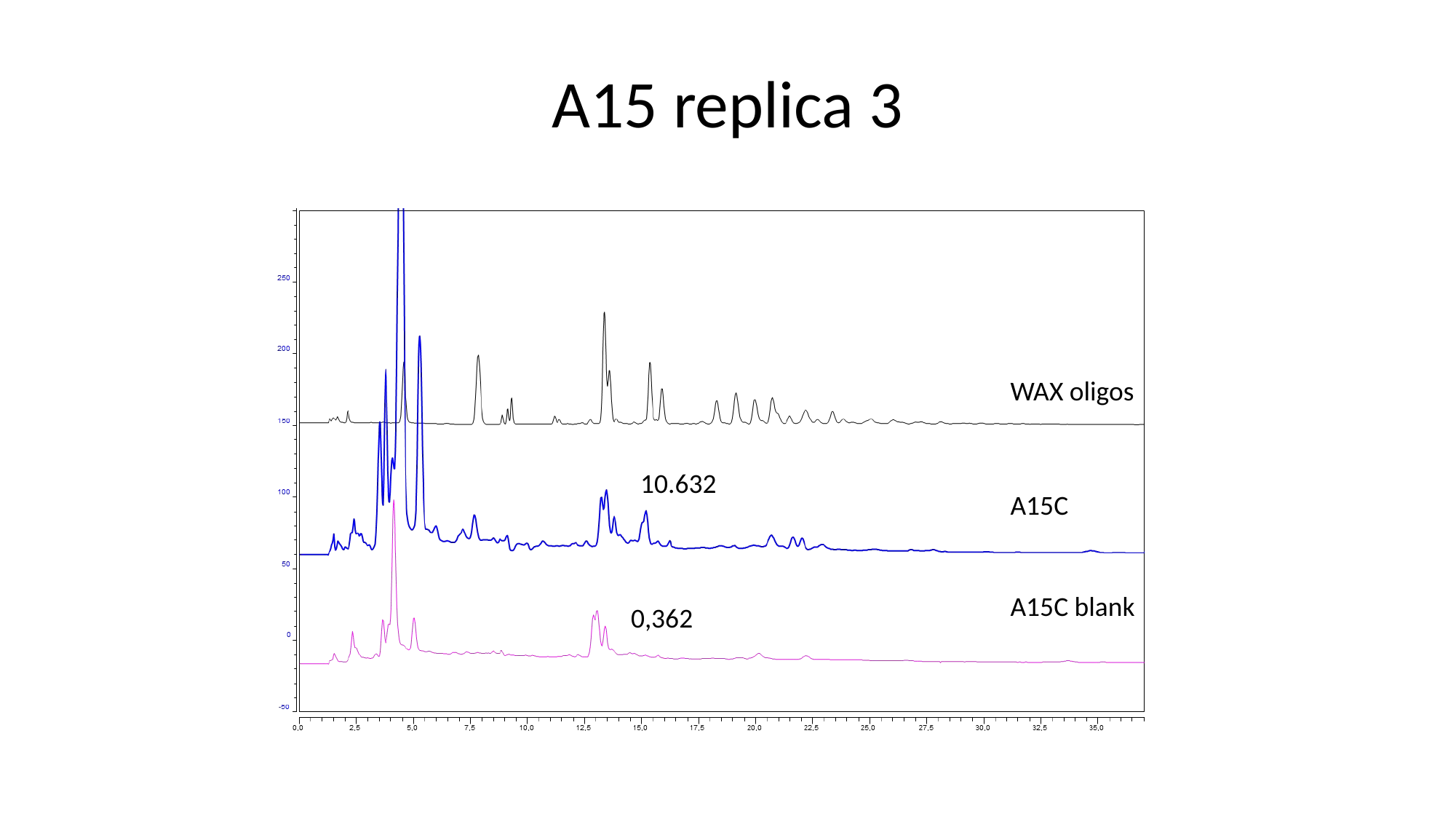

# A15 replica 3
WAX oligos
10.632
A15C
A15C blank
0,362

## Slide 6
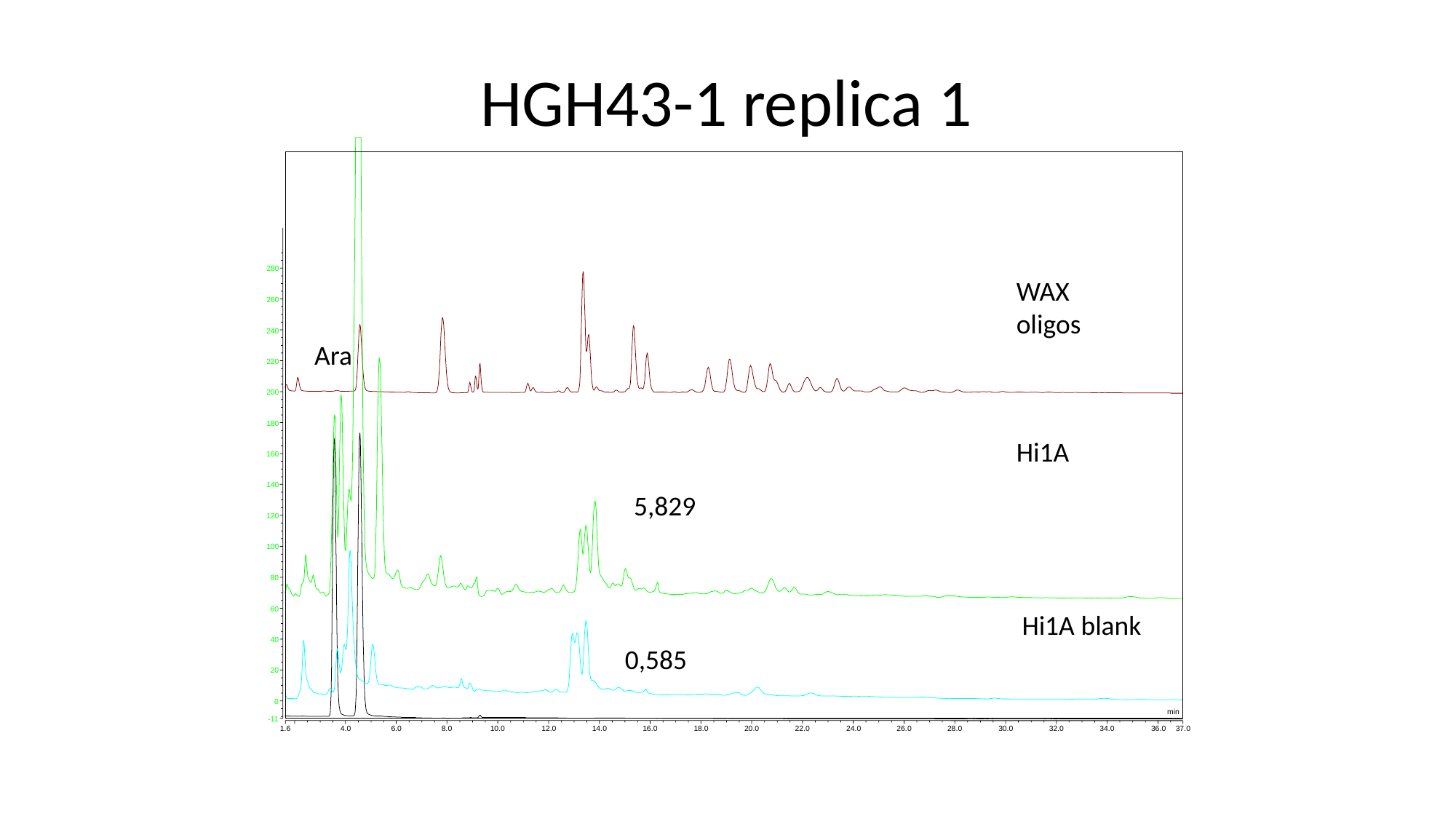

# HGH43-1 replica 1
280
WAX oligos
260
240
Ara
220
200
180
Hi1A
160
140
5,829
120
100
80
Hi1A blank
60
40
0,585
20
0
min
-11
1.6
4.0
6.0
8.0
10.0
12.0
14.0
16.0
18.0
20.0
22.0
24.0
26.0
28.0
30.0
32.0
34.0
36.0
37.0

## Slide 7
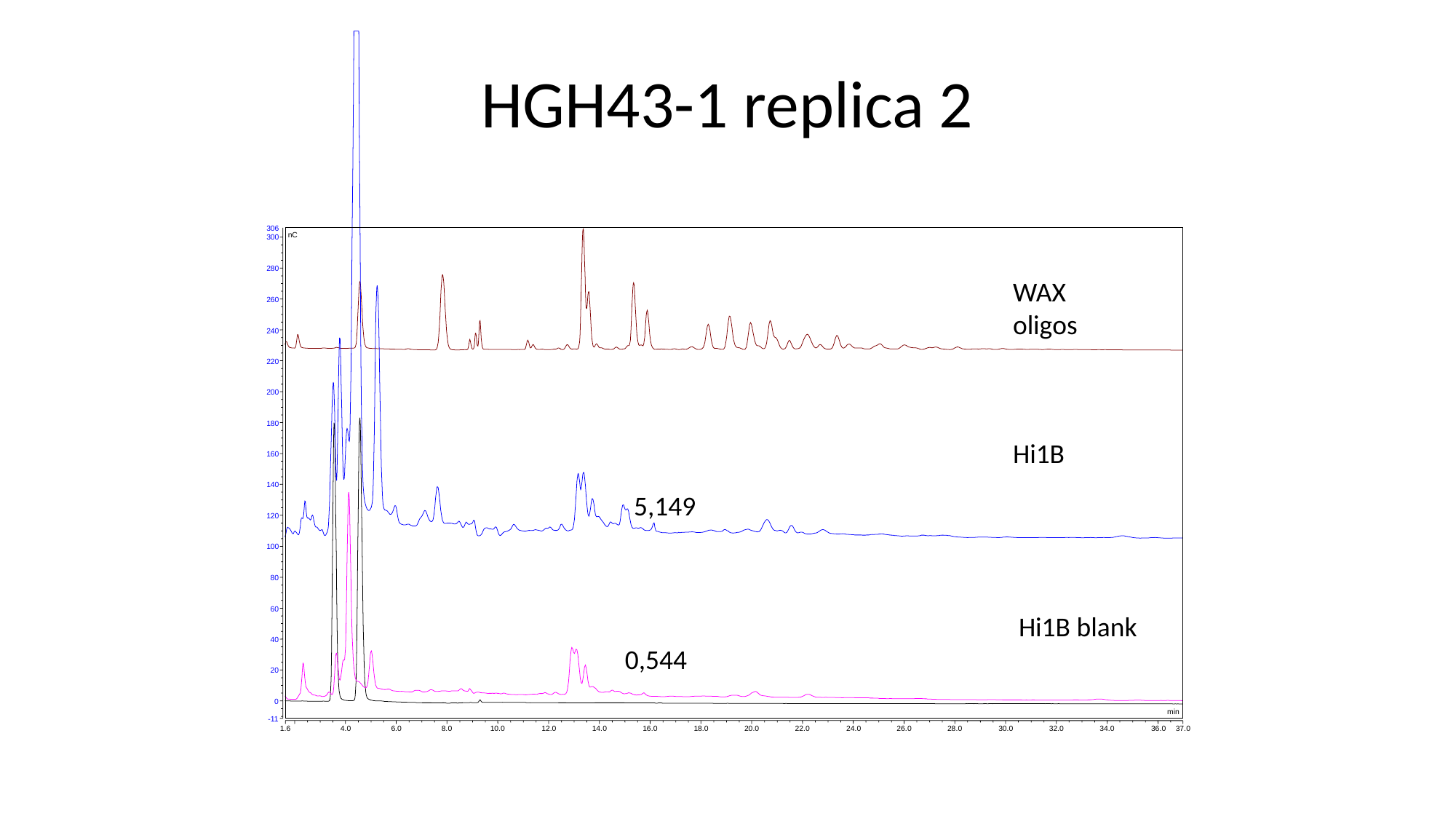

# HGH43-1 replica 2
306
nC
300
280
WAX oligos
260
240
220
200
180
Hi1B
160
140
5,149
120
100
80
60
Hi1B blank
40
0,544
20
0
min
-11
1.6
4.0
6.0
8.0
10.0
12.0
14.0
16.0
18.0
20.0
22.0
24.0
26.0
28.0
30.0
32.0
34.0
36.0
37.0

## Slide 8
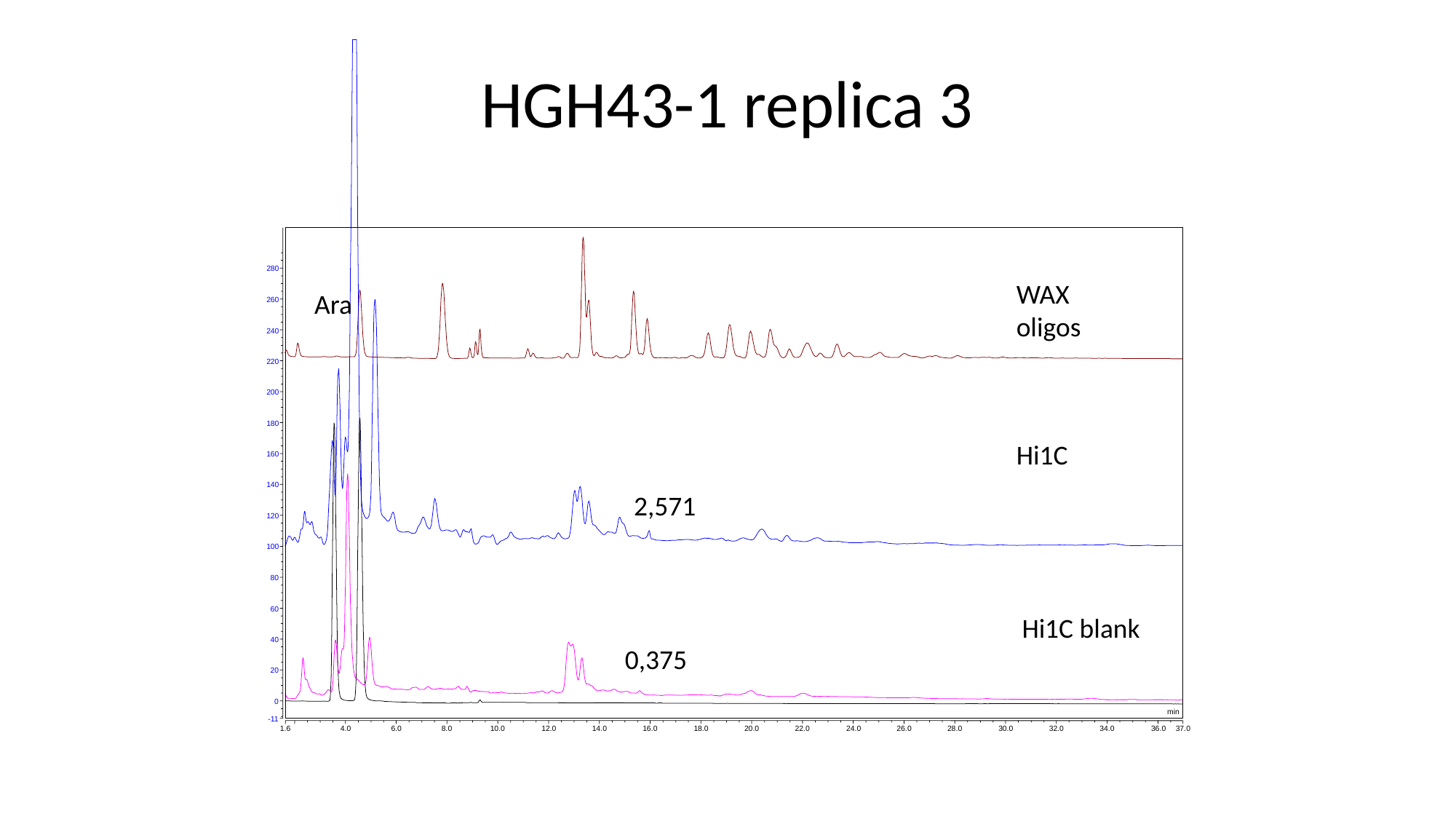

# HGH43-1 replica 3
280
WAX oligos
Ara
260
240
220
200
180
Hi1C
160
140
2,571
120
100
80
60
Hi1C blank
40
0,375
20
0
min
-11
1.6
4.0
6.0
8.0
10.0
12.0
14.0
16.0
18.0
20.0
22.0
24.0
26.0
28.0
30.0
32.0
34.0
36.0
37.0

## Slide 9
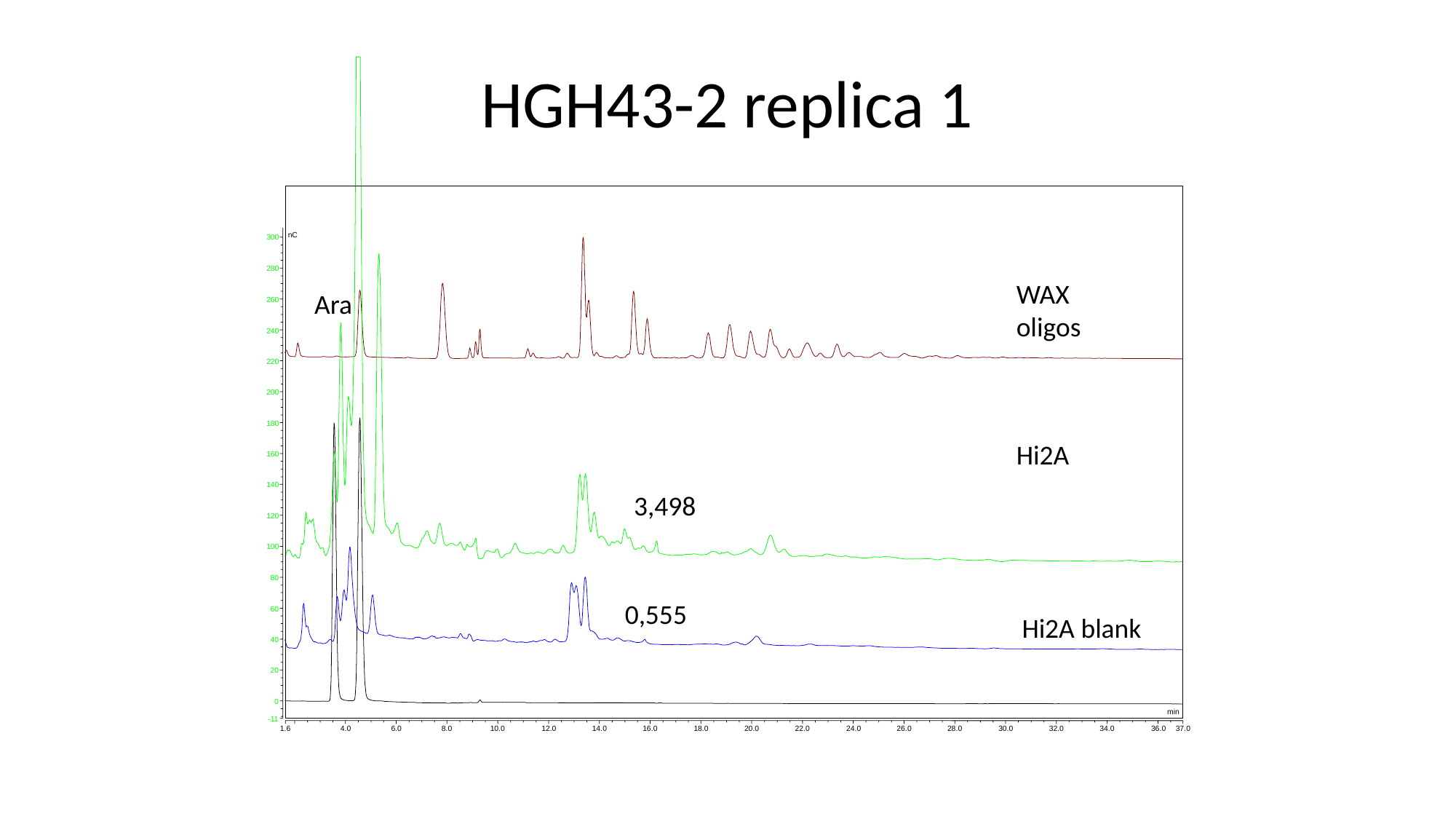

# HGH43-2 replica 1
nC
300
280
WAX oligos
Ara
260
240
220
200
180
Hi2A
160
140
3,498
120
100
80
0,555
60
Hi2A blank
40
20
0
min
-11
1.6
4.0
6.0
8.0
10.0
12.0
14.0
16.0
18.0
20.0
22.0
24.0
26.0
28.0
30.0
32.0
34.0
36.0
37.0

## Slide 10
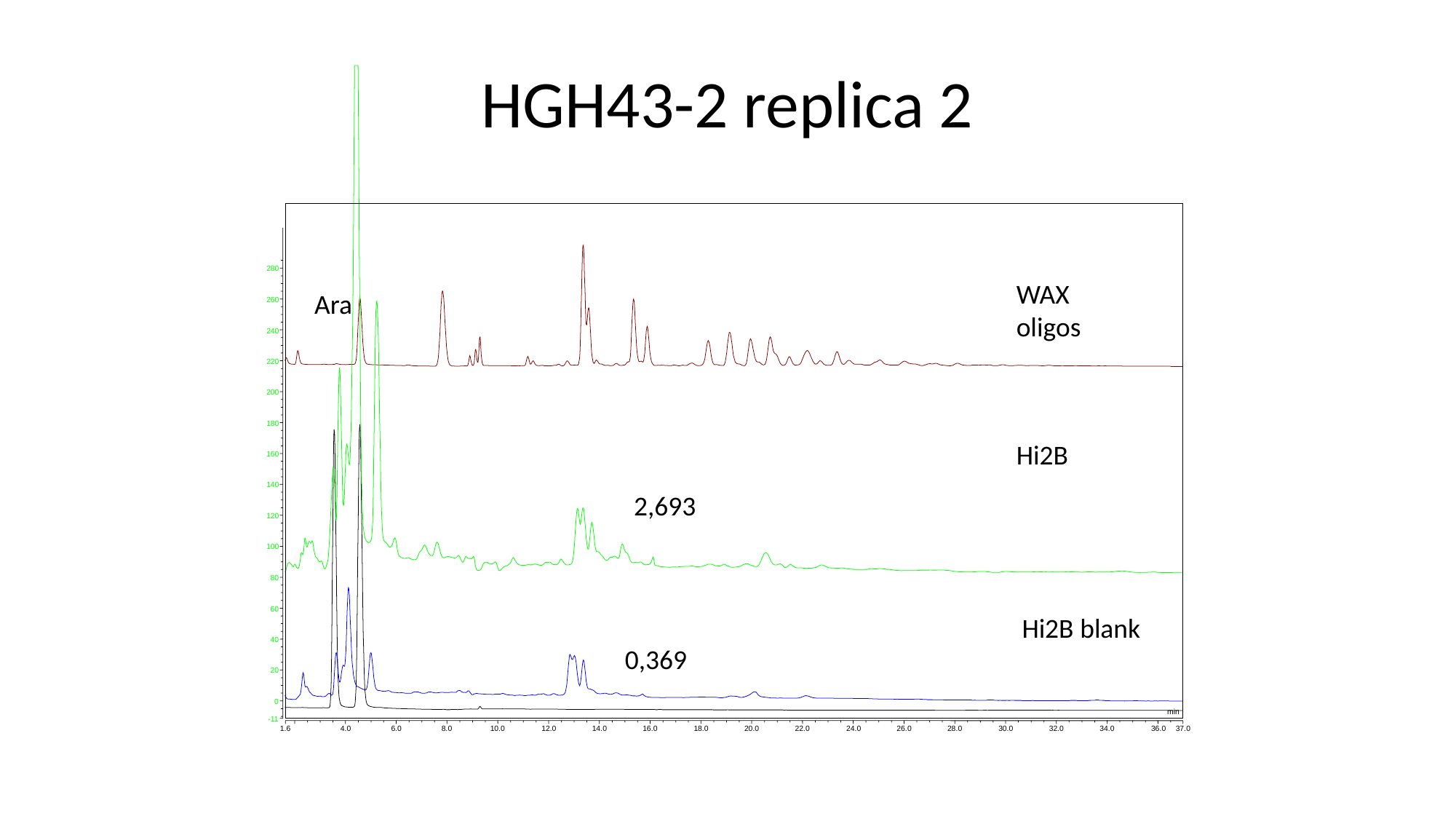

# HGH43-2 replica 2
280
WAX oligos
Ara
260
240
220
200
180
Hi2B
160
140
2,693
120
100
80
60
Hi2B blank
40
0,369
20
0
min
-11
1.6
4.0
6.0
8.0
10.0
12.0
14.0
16.0
18.0
20.0
22.0
24.0
26.0
28.0
30.0
32.0
34.0
36.0
37.0

## Slide 11
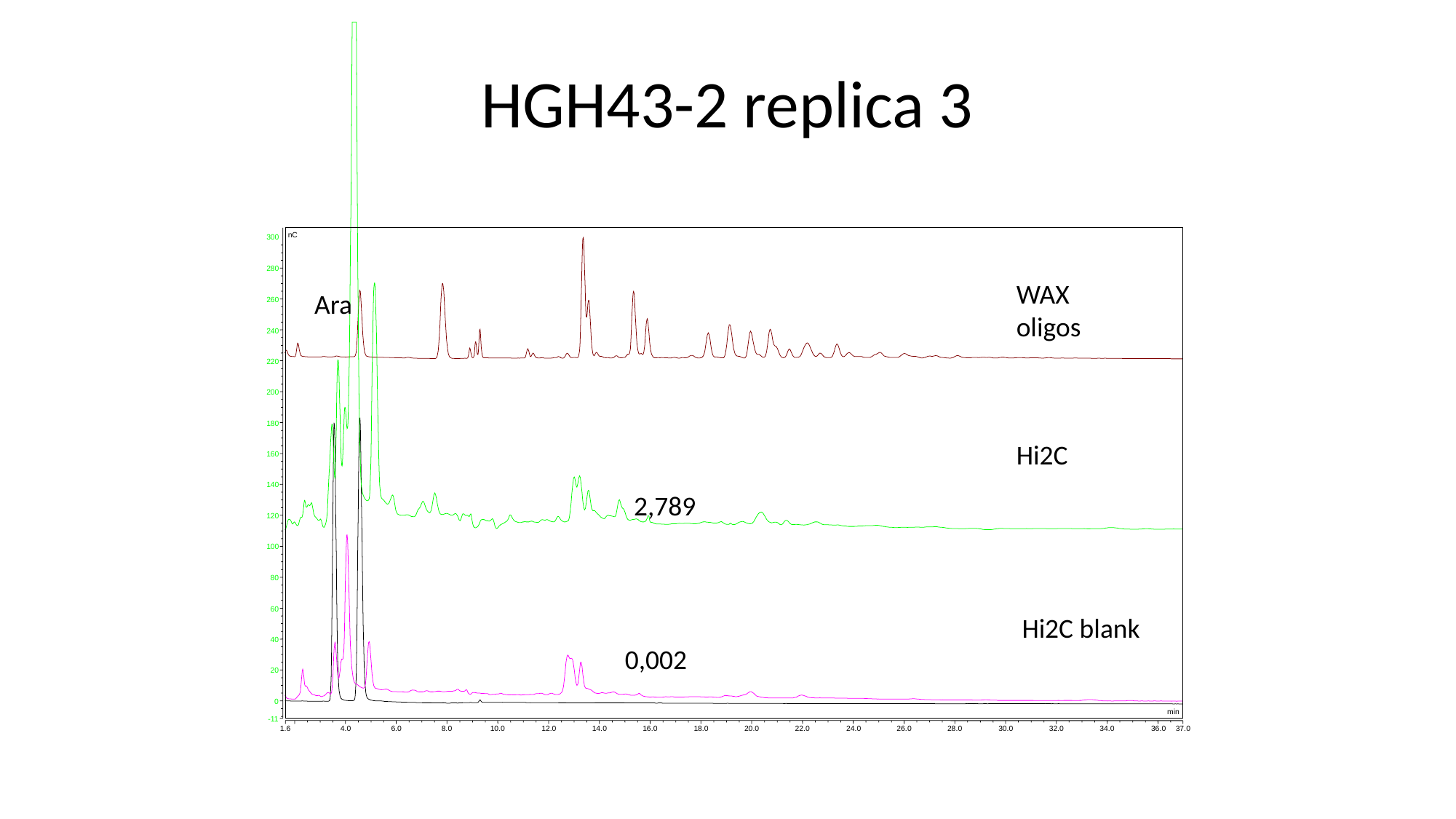

# HGH43-2 replica 3
nC
300
280
WAX oligos
Ara
260
240
220
200
180
Hi2C
160
140
2,789
120
100
80
60
Hi2C blank
40
0,002
20
0
min
-11
1.6
4.0
6.0
8.0
10.0
12.0
14.0
16.0
18.0
20.0
22.0
24.0
26.0
28.0
30.0
32.0
34.0
36.0
37.0
